# Multipoint pacing is associated with improved prognosis and cardiac resynchronization therapy response: MORE-CRT MPP randomized study secondary analyses

**DOI:** 10.1093/europace/euae259

**Published:** 2024-10-04

**Authors:** Leonardo Calò, Ermenegildo De Ruvo, Christof Kolb, Amir Janmohamed, Pedro Marques, Pascal Defaye, Christelle Marquie, Olivier Piot, Andrea Grammatico, Kwangdeok Lee, Wenjiao Lin, Haran Burri, Johannes Sperzel, Bernard Thibault, Christopher Rinaldi, Christophe Leclercq

**Affiliations:** Division of Cardiology, Policlinico Casilino, Rome, Italy; Division of Cardiology, Policlinico Casilino, Rome, Italy; Department of Cardiology, Deutsches Herzzentrum München, Munich, Germany; Department of Cardiology, Rouge Valley Centenary, Toronto, ON, Canada; Cardiology Department, Hospital de Santa Maria, Lisboa, Portugal; Department of Cardiology, Grenoble Alpes University Hospital, Grenoble, France; Department of Cardiology, Centre Hospitalier Régional Universitaire de Lille, Lille, France; Cardiology Department, Centre Cardiologique Du Nord, Saint-Denis, France; Medical Affairs, Abbott, Rome, Italy; Clinical Affairs, Abbott, Plano, USA; Clinical Affairs, Abbott, Plano, USA; Department of Cardiology, Hôpital Cantonal Universitaire de Geneva, Geneva, Switzerland; Kerckhoff Heart and Thorax Center, Kerckhoff Klinik, Bad Nauheim, Germany; Electrophysiology Service Department of Cardiology, Université de Montréal, Montreal, Canada; Department of Cardiology, St Thomas' Hospital, Guy's and St Thomas' NHS Foundation Trust, London, United Kingdom; Service de Cardiologie et Maladies Vasculaires, CHRU Hopital de Pontchaillou, Rennes, France

**Keywords:** Multipoint pacing, MPP, Heart failure, Biventricular pacing, Cardiac resynchronization, Randomized controlled study, Quadripolar left ventricular pacing

## Abstract

**Aims:**

Cardiac resynchronization therapy (CRT) via biventricular (BIV) pacing is indicated in patients with heart failure (HF), reduced ejection fraction, and prolonged QRS duration. Quadripolar leads and multipoint pacing (MPP) allow multiple left ventricle (LV) sites pacing. We aimed to assess the clinical benefit of MPP in patients who do not respond to standard BIV pacing.

**Methods and results:**

Overall, 3724 patients were treated with standard BIV pacing. After 6 months, 1639 patients were considered as CRT non-responders (echo-measured relative reduction in LV end-systolic volume (LVESV) < 15%) and randomized to MPP or BIV. We analysed 593 randomized patients (291 MPP, 302 BIV), who had BIV pacing >97% of the time before randomization and complete 12 months of clinical and echocardiographic data. The endpoint composed of freedom from cardiac death and HF hospitalizations and by LVESV relative reduction ≥15% between randomization and 12 months occurred more frequently in MPP [96/291 (33.0%)] vs. BIV [71/302 (23.5%), *P* = 0.0103], which was also confirmed at multivariate analysis (hazard ratio = 1.55, 95% confidence interval = 1.02–2.34, *P* = 0.0402 vs. BIV). HF hospitalizations occurred less frequently in MPP [14/291 (4.81%)] vs. BIV [29/302 (9.60%), incidence rate ratio = 50%, *P* = 0.0245]. Selecting patients with a large (>30 ms) dispersion of interventricular electrical delay among the four LV lead dipoles, reverse remodelling was more frequent in MPP [18/51 (35.3%)] vs. BIV [11/62 (17.7%), *P* = 0.0335].

**Conclusion:**

In patients who do not respond to standard CRT despite the high BIV pacing percentage, MPP is associated with lower occurrence of HF hospitalizations and higher probability of reverse LV remodelling compared with BIV pacing.

## Graphical Abstract

**Graphical Abstract:**
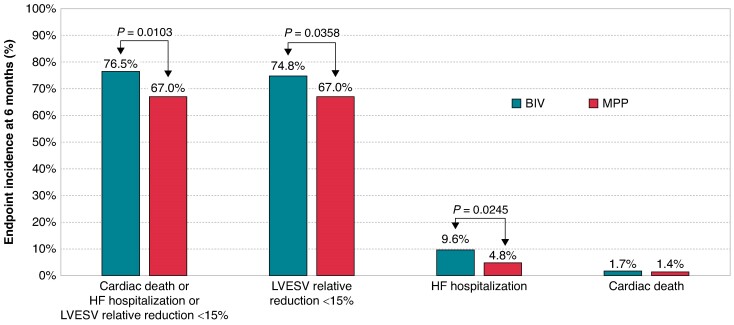

**What's new?**

- Cardiac resynchronization therapy (CRT) via biventricular (BIV) pacing is indicated in patients with heart failure (HF), reduced ejection fraction, and prolonged QRS duration.
- Multipoint pacing (MPP) allows pacing multiple left ventricle (LV) sites with optimal activation times.
- MORE-CRT MPP trial followed 3724 patients treated with standard BIV pacing; at 6 months follow-up, 1639 CRT non-responders [echo-measured LV end-systolic volume (LVESV) relative reduction <15%] were randomized to continue standard BIV pacing or to enable MPP.
- In order to compare standard and optimized biventricular pacing in the best conditions to obtain full CRT benefit (i.e. steady capture of both ventricles), we selected the cohort of 593 patients (302 BIV and 291 MPP) who had BIV pacing for more than 97% of the time before randomization.
- In the 6 months after randomization, MPP patients experienced less HF hospitalizations and more frequently responded to CRT (LVESV relative reduction >15%) compared with BIV patients.

## Introduction

Cardiac resynchronization therapy (CRT) is an established treatment for patients with heart failure (HF), with reduced ejection fraction, and prolonged QRS duration.^1–3^

Quadripolar leads have been added to the CRT armamentarium in 2011 to reach the best LV pacing site.^4^ Then, in 2013, the MultiPoint™ Pacing (MPP) algorithm has been proposed to deliver sequential pacing from two left ventricle (LV) pacing sites.^5–9^ Quadripolar leads and MPP brought the promise to overcome unfavourable coronary venous anatomy, presence of ischemic scars, phrenic nerve stimulation, high capture thresholds, or pacing lead instability. Several observational studies have shown that MPP improves contractility, haemodynamic, and dyssynchrony in acute evaluations^5–9^ and improves mid- and long-term LV reverse remodelling and clinical response compared with biventricular (BIV) pacing.^10–12^ Three randomized studies^13–16^ have compared MPP with standard BIV pacing, with contradictory results. Almusaad *et al*.^13^ showed that MPP provides improved reverse remodelling when programmed at implant. Marques *et al*.^14^ showed that MPP applied on top of standard BIV pacing in patients who responded to CRT further improved LV reverse remodelling and quality of life. On the other hand, the Cardiac Resynchronization Therapy with MultiPoint Pacing (MORE-CRT MPP) trial^15–17^ has not shown superiority of MPP when programmed after 6 months of BIV pacing to reverse LV remodelling in CRT non-responder patients.

It is well known that the degree of response to CRT is highly related to BIV pacing percentage with a minimal value of 97% to obtain a full CRT benefit.^18–21^ We have therefore hypothesized that MPP benefit in the MORE-CRT MPP trial could have been diluted by the fact that some patients received an insufficient CRT therapy, i.e. a low percentage of BIV pacing.^18^

We have therefore performed a secondary analysis of MORE-CRT MPP trial data, selecting the sub-group of patients with high BIV pacing percentage, with the aim of addressing two questions: (i) whether MPP is associated with improved CRT response when applied in optimal conditions (i.e. high biventricular pacing) and (ii) which may be the electrophysiological mechanisms of MPP action.

## Methods

### Study design

The MORE-CRT MPP trial was a prospective, randomized, international multi-centre study (ClinicalTrials.gov Identifier: NCT02006069).

The study was approved by the ethics committee of all participating centres and was conducted in compliance with the Declaration of Helsinki. All patients provided written informed consent.

The study was designed according to two phases. In the first observational phase, all patients were programmed with standard BIV pacing. At 6 months follow-up, echocardiographic measurements were performed and patients without reverse remodelling (echo-measured relative reduction in LV end-systolic volume (LVESV) from baseline to 6 months <15%) were 1:1 randomized in two arms, MPP or BIV pacing.^15–17^ Patients were blinded to the treatment they received in the trial.

An independent Echocardiography Core Laboratory blinded to device programming analyzed the echocardiographic measurements at baseline, 6 months, and 12 months.

### Study participants

The MORE-CRT MPP study enrolled patients with a standard CRT indication according to international guidelines. The study inclusion and exclusion criteria have been previously reported.^15–17^

For the analyses described in this manuscript, we selected the cohort of patients who had BIV pacing percentage >97% in the first 6 months after implant. The hypothesis behind this patient selection is that the benefit of a CRT algorithm, such as MPP, may be detected over standard BIV pacing only when CRT is actually delivered with a steady capture of both right and left ventricles and not when BIV pacing percentage is low, possibly due to AT/AF or frequent PVCs or non-optimal AV pacing delay settings.^18–21^ The choice of the specific pacing percentage cut-off (BIV pacing percentage >97%) was based on the fact that 98% represented the median BIV pacing percentage in the whole population of MORE-CRT MPP and on cut-off values, which in previous studies, were associated with reduced CRT benefits and with higher incidence of HF hospitalizations and of all-cause death.^18–21^

### CRT device and multipoint pacing algorithm

Commercially available MPP-capable CRT-D devices (Unify Quadra MP or Quadra Assura MP, St Jude Medical, Sylmar, CA) and quadripolar LV leads (Quartet™ LV lead, St Jude Medical, Sylmar, CA) were implanted in this study. Implantation was performed according to the standard practice of the individual centres. These devices feature the MultiPoint™ Pacing algorithm that allows to deliver sequential pacing pulses from two LV sites of the same LV lead, potentially capturing a larger area and engaging multiple zones in the long axis of the LV. Two LV pacing vectors (LV1 and LV2) can be selected from the 10 vectors available with the quadripolar systems: six with both cathodes and anodes of the LV lead and four with an LV lead cathode and the right-ventricular coil anode. Interventricular (LV–RV) delay and intraventricular (LV1–LV2) delay are programmable in the range 5–80 and 5–50 ms respectively.

### Device programming

At implant and during the following 6 months, CRT devices were programmed to deliver standard BIV pacing with the LV pacing vector, AV delay, and VV delay settings at physician discretion.

After randomization, CRT devices were programmed to BIV pacing or MPP according to the randomized arm. The specific programming of patients randomized to the MPP arm, in terms of which pacing vectors were selected from the 10 vectors available with the quadripolar lead systems, was decided by each study investigator according to patient-tailored considerations, such as interventricular electrical delays, pacing thresholds and the occurrence of phrenic nerve stimulation, and according to the possibility to program a wide separation (≥30 mm) between the two cathodal electrodes. Also in MPP patients, the following delays were suggested: LV1–LV2 delay = 5 ms and LV2–RV delay = 5 ms.

### Analysis objectives

We have performed a secondary analysis of MORECRT MPP trial data, selecting the sub-group of patients with high BIV pacing percentage, with the aim of addressing two questions: (i) whether MPP is associated with improved CRT response when applied in optimal conditions (i.e. high biventricular pacing) and (ii) which may be the electrophysiological mechanisms of MPP action.

### Study endpoints

The primary endpoint of our analysis was a composite of cardiac death, HF hospitalizations, and LVESV relative reduction less than 15%. Unless otherwise specified, patients were considered responders to CRT if they were free from cardiac death and HF hospitalizations at 6 months after randomization and if LVESV relative reduction was greater or equal to 15% when comparing echo measurements performed at randomization and at 6 months after randomization.

Secondary endpoints comprised the single components of the composite endpoint. HF hospitalizations were defined as hospitalizations or emergency department visits, with HF as main diagnosis and lasting ≥24 h, or hospitalizations or emergency department visits, with HF as main diagnosis and lasting <24 h, but requiring intravenous administration of inotropes or diuretics.

Device diagnostics allowed to measure for each patient the interventricular electrical delay as the time from RV sensing to LV activation (RV–LV delay) during intrinsic rhythm for each of the four electrodes of the quadripolar LV lead (D1, M2, M3, and P4). Based on these four RV–LV delays, we estimated for each patient the dispersion (ΔLV) of interventricular electrical delay along the LV lead axis as the longer time difference between any couple of RV–LV delay of the four LV electrodes.

### Intention to treat analysis

Our main analysis was performed according to the intention to treat approach, i.e. according to the treatment to which patients were randomized. The analysed patient population includes patients who were randomized into either the MPP or BIV arm and completed study visits and had complete echocardiographic data or reached the study clinical endpoints, i.e. cardiac death or HF hospitalization before the last follow-up visit. We also evaluated the primary endpoint according to the on-treatment analysis approach.

### Statistical analysis

All reported characteristics were described using summary statistics. Continuous variables were expressed as means and standard deviations or median and interquartile range (IQR), as appropriate. Categorical variables were expressed as counts and percentages. Comparison of baseline characteristics between the two randomized groups was performed with Student *t*-test, Wilcoxon signed-rank test, or χ^2^ proportion test as appropriate.

All data were analysed according to the intention-to-treat principle. The analysis set included all the patients randomized who had complete clinical and echocardiographic data up to 12 months follow-up.

The risk of clinical outcomes in the two randomization arms was evaluated by estimating the incidence rate ratio (IRR) as the ratio of the number of events in one arm to the number of events in the other arm and using the Kaplan–Meier method and comparing the cumulative hazard curves by means of the log-rank test.

Logistic regression models were implemented in order to find independent predictors of CRT response at 12 months. Hazard ratios and 95% confidence interval (CI) were also calculated. The probability of CRT response between MPP and BIV in patient sub-groups according to baseline characteristics was derived from multivariable logistic regression models and compared using χ^2^ test.

No imputation of missing data was performed.

Statistical tests were based on a two-sided significance level of 0.05.

Analyses were conducted using SAS software (version 9.4, SAS Institute Inc., USA).

## Results

**Figure 1:**
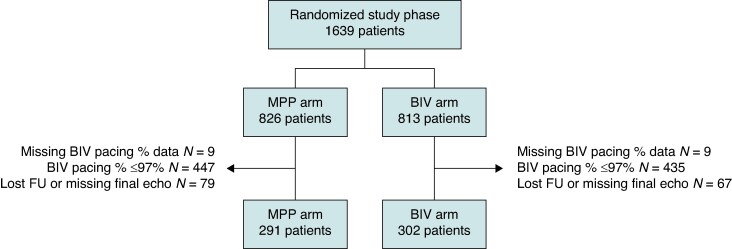
Study flow chart.

In the first observational phase of the study, 3724 patients completed the 6 months follow-up with complete echo measurements data, from which 1639 patients were considered as CRT non-responders and were randomized to MPP or BIV. The median (quartile range) of the BIV pacing percentage during the first 6 months after implant was 98% (93–100%). As shown in the study diagram (*Figure 1*), for the analyses described in this manuscript, we selected the 593 patients who had complete echo measurement data and high BIV pacing percentage (i.e. >97%). The baseline characteristics of these patients are described in *Table 1*. Importantly, patients’ characteristics in this sub-group did not difer from those of the whole population, which was randomized in the study. Overall the CRT device was a defibrillator in 519/593 (87.5%) patients and a pacemaker in 74/593 (12.5%) patients, with no statistical differences between the two randomization arms.

**Table 1:** Baseline demographics of MORE-CRT MPP study patients who were classified as non-responder despite high BIV pacing percentage (whole cohort and MPP and BIV groups)

	Whole cohort subjects (N = 593)	MPP subjects (N = 291)	BIV subjects (N = 302)	*P* value
Age (years) Mean ± standard deviation (N)	67 ± 10 (593)	67 ± 10 (291)	68 ± 11 (302)	0.5879
Gender, % (n/N)				
Female	22.6% (134/593)	20.3% (59/291)	24.8% (75/302)	0.1844
Male	77.4% (459/593)	79.7% (232/291)	75.2% (227/302)	
New York Heart Association Class, % (n/N)				
II	50.8% (301/593)	50.9% (148/291)	50.7% (153/302)	0.9760
III	46.9% (278/593)	47.1% (137/291)	46.7% (141/302)	
IV	2.2% (13/593)	2.1% (6/291)	2.3% (7/302)	
Ischemic cardiomyopathy, % (n/N)	54.0% (320/593)	54.0% (157/291)	54.0% (163/302)	0.9958
Medical history, % (n/N)				
Hypertension	62.7% (372/593)	63.6% (185/291)	61.9% (187/302)	0.6772
Hypercholesterolemia	43.0% (255/593)	46.4% (135/291)	39.7% (120/302)	0.1017
Myocardial infarction	37.9% (225/593)	39.9% (116/157)	36.1% (109/163)	0.1698
Diabetes mellitus	37.6% (223/593)	37.1% (108/291)	38.1% (115/302)	0.8082
Renal disease	19.6% (116/593)	20.6% (60/291)	18.5% (56/302)	0.5241
Chronic obstructive pulmonary disease	9.6% (57/593)	9.6% (28/291)	9.6% (29/302)	0.9936
Stroke	3.9% (23/593)	4.5% (13/291)	3.3% (10/302)	0.4661
TIA	3.2% (19/593)	4.1% (12/291)	2.3% (7/302)	0.2119
Medication, % (n/N)				
ACE/ARBs	87.5% (519/593)	86.6% (252/291)	88.4% (267/302)	0.5043
Beta-blockers	85.7% (508/593)	86.3% (251/291)	85.1% (257/302)	0.6882
Diuretics	76.6% (454/593)	77.3% (225/291)	75.8% (229/302)	0.6681
ACE	61.4% (364/593)	60.5% (176/291)	62.3% (188/302)	0.6580
Statins	61.0% (362/593)	62.5% (182/291)	59.6% (180/302)	0.4629
Aldosterone antagonist	37.3% (221/593)	39.5% (115/291)	35.1% (106/302)	0.2658
Antiplatelets	56.2% (333/593)	56.0% (163/291)	56.3% (170/302)	0.9457
Anticoagulants	30.9% (183/593)	30.6% (89/291)	31.1% (94/302)	0.8865
ARBs	29.5% (175/593)	30.6% (89/291)	28.5% (86/302)	0.5738
Anti-arrhythmics	20.4% (121/593)	20.3% (59/291)	20.5% (62/302)	0.9386
Calcium channel blockers	7.9% (47/593)	7.6% (22/291)	8.3% (25/302)	0.7463
Nitrates	7.6% (45/593)	7.2% (21/291)	7.9% (24/302)	0.7370
Ventricular conduction disease, % (n/N)				
Left bundle branch block	60.2% (292/485)	60.7% (145/239)	59.8% (147/246)	0.8372
Intra ventricular conduction delay	26.4% (128/485)	24.3% (58/239)	28.5% (70/246)	0.2955
Right bundle branch block	10.1% (49/485)	11.7% (28/239)	8.5% (21/246)	0.2455
Left anterior fascicular block	6.8% (33/485)	5.9% (14/239)	7.7% (19/246)	0.4146
QRS interval Mean ± standard deviation (N)	156 ± 24 (544)	156 ± 24 (261)	156 ± 25 (283)	0.9016
Left ventricle end systolic volume (ml) Mean ± standard deviation (N)	155 ± 67 (593)	155 ± 64 (291)	156 ± 69 (302)	0.9002
Left ventricle ejection fraction (%) Mean ± standard deviation (N)	27 ± 8 (593)	27 ± 8 (291)	27 ± 7 (302)	0.7793

ACE, Angiotensin-converting enzyme; ARB, Angiotensin II receptor blocker.

Out of 291 patients randomized to MPP arm, 279 (95.9%) were programmed according to randomization, and, in particular, 140 patients were programmed with a wide separation (≥30 mm) between the two LV electrodes; overall, 12 patients randomized to MPP were actually programmed with standard biventricular pacing.

Out of 302 patients randomized to BIV arm, five were actually programmed enabling the MPP algorithm, and one patient was programmed with RV pacing only.

In the 6 months after randomization, the median (interquartile range) percentage of biventricular pacing was 100% (98–100%) in both randomized groups.

Heart failure medications did not significantly change comparing randomization visit and 12 months follow-up visit; also, there were no statistical differences comparing medications in the two randomized arms at different study visits.

### Is MPP associated with clinical benefit compared with BIV?

The proportion of patients considered as CRT responder, because they were free from cardiac death and HF hospitalizations and because CRT provided a positive LV reverse remodelling (LVESV relative reduction ≥15%), was higher in MPP patients (96/291 (33.0%)) compared to BIV patients (71/302 (23.5%), *P* = 0.0103), according to the intention-to-treat analysis. Following the on-treatment analysis approach, CRT responders were 91/284 (32.0) in MPP patients and 76/308 (24.7%) in BIV patients (*P* = 0.0466).

HF hospitalization occurred less frequently in MPP patients [4.81% (14/291)] compared with BIV [9.60% (29/302), IRR = 50%, *P* = 0.0245].

Cardiac death or HF hospitalizations occurred less frequently in MPP patients [5.84% (17/291)] compared with BIV [11.26% (34/302), IRR = 48%, *P* = 0.0187].

LV reverse remodelling, in terms of LVESV relative reduction ≥15% between randomization and 6 months after, was more frequently observed in MPP patients [96/291 (33.0%)] compared to BIV [76/302 (25.2%), *P* = 0.0358].

**Figure 2:**
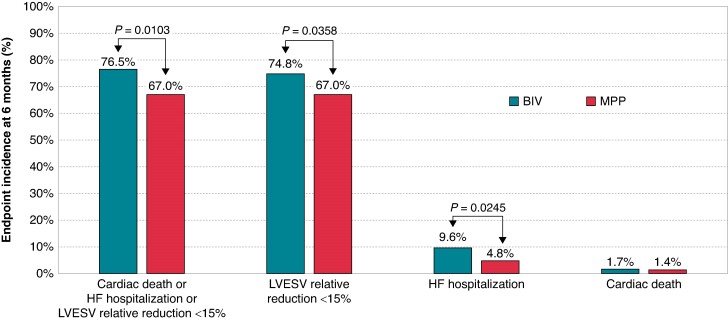
Occurrence of cardiac death, HF hospitalizations, failed reverse remodelling, and failed CRT response.

Occurrence of cardiac death, HF hospitalizations, failed reverse remodelling, and failed CRT response in the 6 months after randomization are shown in *Figure 2*.

**Figure 3:**
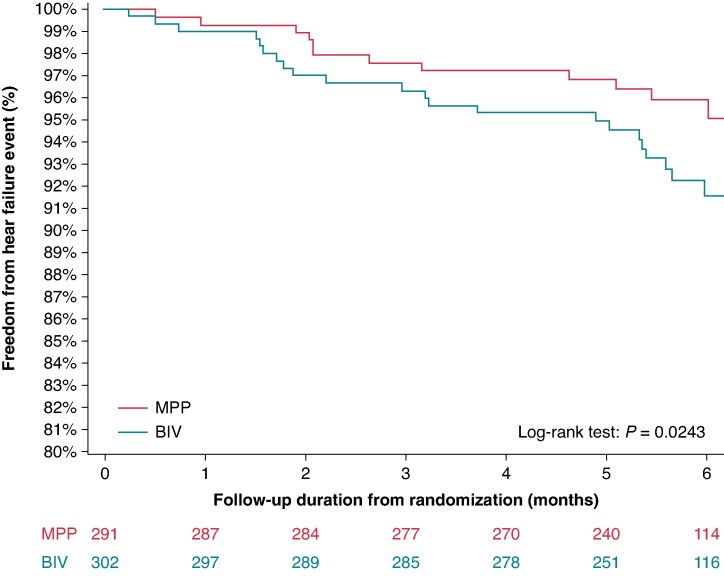
Kaplan–Meier incidence of heart failure hospitalizations in MPP and BIV arms.

Kaplan–Meier incidence of HF hospitalizations is shown in *Figure 3*.

Kaplan–Meier incidence of HF hospitalizations or cardiac death is shown in Supplementary Data Figure*A*.

Multivariate analysis confirmed that the probability of being a CRT responder because of freedom from cardiac death or HF hospitalizations and because of positive LV reverse remodelling was higher in patients randomized to MPP compared to BIV with a hazard ratio = 1.55 (95% CI = 1.02–2.34, *P* = 0.0402), as shown in *Table 2*, when taking into account the most important patient characteristics.

**Table 2:** Logistic regression univariate and multivariate analysis for CRT response defined as freedom from cardiac death or HF hospitalizations and as LV reverse remodelling

	Univariate analysis			Multivariable analysis		
Parameters	Hazard ratio [95% CI]	*P* value	Sample size	Hazard ratio [95% CI]	*P* value	Sample size
Age	0.99 [0.97, 1.01]	0.2819	593	0.99 [0.97, 1.01]	0.4082	480
Ischaemic vs. non-Ischaemic	**0.52 [0.36, 0.76]**	**0**.**0005**	**593**	**0.64 [0.41, 0.99]**	**0**.**0441**	**480**
LBBB vs. non-LBBB	1.60 [1.05, 2.44]	0.0289	485	1.51 [0.96, 2.37]	0.0765	480
6 M LVEF ≥29% vs. <29%	0.87 [0.61, 1.25]	0.4628	593	1.08 [0.65, 1.78]	0.7716	480
6 M LVESV ≥150.5 vs. <150.5	1.38 [0.96, 1.98]	0.0860	593	1.48 [0.89, 2.48]	0.1322	480
NYHA I/II vs. NYHA III/IV	1.23 [0.86, 1.76]	0.2632	592	1.31 [0.86, 2.00]	0.2010	480
QRS ≥150 ms vs. <150 ms	1.41 [0.95, 2.10]	0.0888	544	1.10 [0.70, 1.73]	0.6776	480
Female vs. male	1.14 [0.74, 1.74]	0.5521	593	1.05 [0.63, 1.75]	0.8471	480
MPP vs. BIV	**1.55 [1.08, 2.23]**	**0**.**0173**	**593**	**1.55 [1.02, 2.34]**	**0**.**0402**	**480**

CI, Confidence interval; LBBB, Left bundle branch block; LVEF, Left ventricle ejection fraction; LVESV, Left ventricle end systolic volume; NYHA, New York Heart Association; MPP, MultiPoint pacing group; BIV, Biventricular pacing group. Bold font was used to outline parameters which were identified as significantly associated with CRT response.

Sub-group analyses performed with hypothesis generation intentions are shown in Supplementary Data Table*A* and Figure*B*.

### Which electrophysiological mechanism may be behind MPP action compared with BIV?

**Figure 4:**
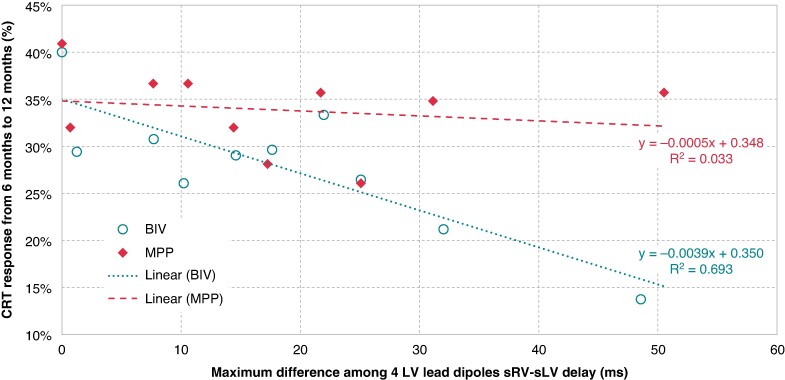
CRT response in terms of proportion of patients with LVESV relative reduction >15%, between 6 months (randomization) and 12 months of visits, as a function of interventricular electrical delay dispersion among the four LV dipoles in the randomized study population.

MPP has been proposed as an improved pacing modality for its capability to provide sequential pacing pulses from two LV sites and to capture a larger area of the LV. We tested the hypothesis that MPP could be better than BIV in patients with a large dispersion of interventricular electrical delay in different zones of the LV. The dispersion of interventricular electrical delay ΔLV had a median of 16 ms (interquartile range 8–24 ms). *Figure 4* shows the proportion of patients who responded to CRT after randomization between BIV and MPP as a function of ΔLV. In BIV patients, CRT response had a decreasing trend as a function of increased ΔLV, while in MPP patients, CRT response did not depend on ΔLV. While MPP showed a superior response in the overall population, its superiority was especially associated with patients with large ΔLV, in particular, it was CRT response in MPP patients with ΔLV > 30 ms that was significantly higher [18/51 (35.3%)] compared to BIV patients [11/62 (17.7%), *P* = 0.0335]. Interestingly, in the whole population of patients who completed the first 6 months of the study before randomization, who were paced with standard BIV pacing, CRT response as a function of ΔLV (see Supplementary data Figure*C*) had a decreasing trend very similar to that found in BIV patients after randomization, confirming in a much larger population that dispersion of interventricular electrical delay along the LV lead axis is an important parameter for CRT optimization.

## Discussion

Cardiac resynchronization therapy for a selected population of HF patients is a well-established treatment with 20 years of history^1–3^ and is an evolving field of research.^22^ Multipoint pacing is an innovative feature in CRT exploiting one of the advantages of the quadripolar leads widely used in routine care. The MORE-CRT MPP trial is the largest randomized study dedicated to assess the potential benefit of MPP in CRT response. The MORE-CRT MPP primary analysis published by Leclercq *et al*.^17^ evaluated CRT response in terms of echocardiographic LV reverse remodelling (LVESV relative reduction >15%) and showed that (i) at 6 months follow-up, about 40% of patients did not respond to standard BIV pacing; (ii) about 30% of them subsequently showed a echocardiographic reverse remodelling at 12 months; and (iii) there was no difference in patient outcomes between MPP and BIV. The last result, neutral in the comparison of MPP and standard BIV pacing, was unexpected given the fact that MPP was associated with superior CRT response in several observational studies^5–12^ and two previous randomized studies.^13,14^ Leclercq *et al*.^17^ suggested that patients with biventricular pacing percentage ≥99% along the entire 12 months study could benefit from MPP. We therefore hypothesized that, in the MORE-CRT MPP trial, specific patients characteristics, such as AT/AF or frequent PVCs, and other conditions, e.g. non-optimal AV delay settings, might have reduced the dose of BIV pacing and, therefore, might have decreased the overall benefit of MPP and/or might have diluted the capability of the study to measure MPP superiority over standard BIV pacing. Therefore, we designed a new retrospective analysis, which was different from the one already performed by Leclercq *et al*.^17^ for the following reasons: (i) we chose the biventricular pacing percentage cut-off as ≥98% based on the median value of pacing percentage in the first 6 months, instead of ≥99% along the entire 12 months; and (ii) we considered CRT response as an endpoint composed not only by LV reverse remodelling, but also by freedom from HF hospitalizations and cardiac death. This definition of the CRT response may improve the capability to assess new therapies’ impact on clinical outcomes and to measure disease modification, as recently proposed by several authors.^23,24^

### Main study findings

Our results show that (i) overall MPP is associated with improved CRT response compared with standard BIV pacing, and (ii) the improved reverse LV remodelling observed in MPP patients is associated with the possibility to capture larger areas of LV in patients with large dispersion of interventricular electrical delay.

### Is MPP associated with clinical benefit compared with BIV?

Our analyses show that when CRT can be delivered with high doses of BIV pacing (>97%), MPP is associated with a lower risk of the endpoint composed by cardiac death, HF hospitalizations, or failed LV reverse remodelling (*Figure 2* and *Table 2*). In particular, MPP is associated with significant reductions of HF hospitalizations, as shown in *Figures 2* and *3*, with an absolute reduction of 4.8%, and a relative reduction 50%. These percentages could be interpreted as clinically relevant when considering that the follow-up period after randomization was only 6 months.

It is also important to look at LV reverse remodelling data since the MORE-CRT MPP trial primary endpoint was the LVESV relative reduction ≥15% between randomization and 6 months after.^15^ The study sample size estimation assumed that 20% of subjects, who were non-responders in the control group, would have become responders at 12 months compared to baseline, and that this proportion would be 30.5% for the subjects with 6 months of MPP treatment. To detect this 10.5% difference between the two groups at a one-sided significance level of 2.5%, the study sample size estimation required at least 536 subjects, 268 subjects in each group, with analysable data, to achieve a power of 80%. Our data on patients with high biventricular pacing percentage show that reverse LV remodelling was more frequently observed in MPP patients [96/291 (33.0%)] compared to BIV [76/302 (25.2%), *P* = 0.0358], with 33% and 25.2% percentages being very similar to those (30.5% and 20%) we initially considered in the study sample size calculation.

These findings confirm the results of previous observational studies^5–12^ and randomized studies,^13,14^ which showed significant benefits of MPP on haemodynamic and echocardiographic responses and on clinical outcomes and suggest that MPP may be a relevant clinical option in patients who do not respond to standard CRT despite high BIV pacing percentage.

### Which electrophysiological mechanism may be behind MPP action compared with BIV?

MPP has been proposed to improve CRT response based on several possible electrophysiological mechanisms. In particular, by pacing specific LV zones and/or capturing a larger LV area, MPP algorithm may be beneficial by (i) reducing intraventricular dyssynchrony, promoting a more coordinated LV contraction, and reducing the total time required for LV activation, (ii) improving the timing of left and right ventricular contraction relative to atrial contraction and therefore optimizing LV filling, (iii) avoiding ischaemic scars and therefore bypassing regions with slow or blocked electrical conduction, and (iv) facilitating the fusion of RV-intrinsic, RV-paced and LV-paced depolarization wavefronts. In LBBB patients or intraventricular conduction delay (IVCD) patients, when they have large QRS, but intact RV conduction, MPP may play a synergistic role with AV delay optimization techniques, both manual or via automatic algorithms.^25,26^

According to these mechanisms, MPP might be associated with improved response in specific patient subgroups, such as ischemic patients or patients with large QRS or IVCD patients.

Our sub-group analyses were definitely not powered to accurately test these hypotheses; anyhow, our data (see Supplementary Data Table*A* and Figure*B*) indeed suggest that MPP may be associated with improved CRT response in ischemic patients, patients with large QRS width, and IVCD patients.

We also aimed to evaluate MPP action mechanisms compared with pacing from a single LV site in patients with large dispersion in interventricular electrical delays along the LV lead axis. Our data show that the proportion of patients who respond to CRT, in terms of LV reverse remodelling, with standard biventricular pacing has a decreasing trend as a function of dispersion of interventricular electrical delay in different zones of the LV (*Figure 4* and Supplementary Data Figure*C*). As expected, the trend of CRT response as a function of ΔLV in BIV patients was very similar in the whole study population in the first 6 months of the study (see Supplementary Data Figure*C*) and in patients who were randomized to BIV pacing in the second 6 months of the study (*Figure 4*), with the only difference being the absolute CRT response, which was lower in the randomized group because those patients were non-responders in the first 6 months. Importantly, our data show that CRT response (LV reverse remodelling) in MPP patients does not depend on dispersion in interventricular electrical delays and that CRT response is higher with MPP compared with BIV (35.3% vs. 17.7%, *P* = 0.0335) in patients with large ΔLV (>30 ms). We selected the value of 30 ms as a cut-off for large dispersion of interventricular electrical delay along the LV lead axis based on our observations. Other authors^27^ also suggested that interventricular electrical delay dispersion may impact CRT response and indicated a ΔLV > 20 ms as a clinically relevant threshold to consider large the dispersion along the LV lead axis.

### Clinical implications

Our study results suggest that MPP could be considered as a second-line therapy in CRT non-responders despite a high BIV pacing. The observation that MPP is associated with improved CRT response in patients with large dispersion of interventricular electrical delays, as measured by the device from the four LV lead dipoles, suggests that, in the future, device data could feed artificial intelligence algorithms to guide optimal device programming based on electrogram data.^28^

### Study limitations

We performed a retrospective analysis of a randomized controlled trial; therefore, the findings of our analyses should be interpreted as exploratory and proposed as generating the hypothesis, rather than proving that programming MPP provides benefit in patients otherwise not responding to standard BIV pacing despite a high percentage of BIV pacing.

We recognize possible limitations due to the retrospective nature of our analyses; anyhow,, we are confident about the strength and solidity of our analyses for the following reasons: (i) the study had a randomized design; (ii) the compared randomized groups were not different in terms of baseline characteristics; (iii) patient characteristics in the sub-group of patients with high biventricular pacing, which is the cohort analysed in this secondary study, did not differ from the characteristics of the whole population, which was randomized in the main study; (iv) the retrospective analysis was performed according to a statistical plan defined before accessing the data, with prespecified analysis endpoints and with a predefined patient cohort, selected on the basis of high pacing percentage; and (v) we confirmed our findings with multivariate logistic regression analyses.

We did not analyse data according to specific LV lead sites or to specific device settings, such as LV pacing vector, AV delay, or VV delay, for two reasons: (i) we wanted to assess the possible value of MPP vs. BIV pacing in a CRT population as general as possible, knowing that variability naturally occurs as for LV pacing site and device programming; and (ii) we thought that our cohort sample would not have given appropriate statistical power to further study sub-groups of patients.

Our results apply to the studied population of CRT patients, who did not respond to standard BIV pacing despite high pacing percentage. As a consequence, our results are not generalizable to all CRT patients.

## Conclusions

Our study suggests that, in patients who do not respond to CRT despite a high percentage of biventricular pacing, MPP is associated with lower occurrence of HF hospitalizations and higher probability of reverse LV remodelling compared with standard BIV pacing. Our findings also suggest that one the mechanisms of MPP benefit is the possibility to capture larger areas of LV in patients with large dispersion of interventricular electrical delay.

## Supplementary Material

euae259_Supplementary_Data

## Data Availability

The data underlying this article were provided by Abbott Laboratories. Data will be shared on request to the corresponding author with permission of Abbott Laboratories.
