# Correction to: Multipoint pacing is associated with improved prognosis and cardiac resynchronization therapy response: MORE-CRT MPP randomized study secondary analyses

**DOI:** 10.1093/europace/euaf022

**Published:** 2025-02-18

This is a correction to: Leonardo Calò *et al.*, Multipoint pacing is associated with improved prognosis and cardiac resynchronization therapy response: MORE-CRT MPP randomized study secondary analyses, *EP Europace*, Volume 26, Issue 11, November 2024, https://doi.org/10.1093/europace/euae259

In the originally published version, author names were presented incorrectly and have been corrected.

