# Supplementary material for: Multipoint pacing is associated with improved prognosis and cardiac resynchronization therapy response: MORE-CRT MPP randomized study secondary analyses
Source: Europace. 2024 Oct 4;26(11):euae259. doi: 10.1093/europace/euae259 (PMC11572720; doi:10.1093/europace/euae259)
Supplement: euae259_Supplementary_Data [file euae259_supplementary_data.docx]

**Supplementary data**

***Figure A - Kaplan-Meier incidence of heart failure hospitalizations or cardiac death***


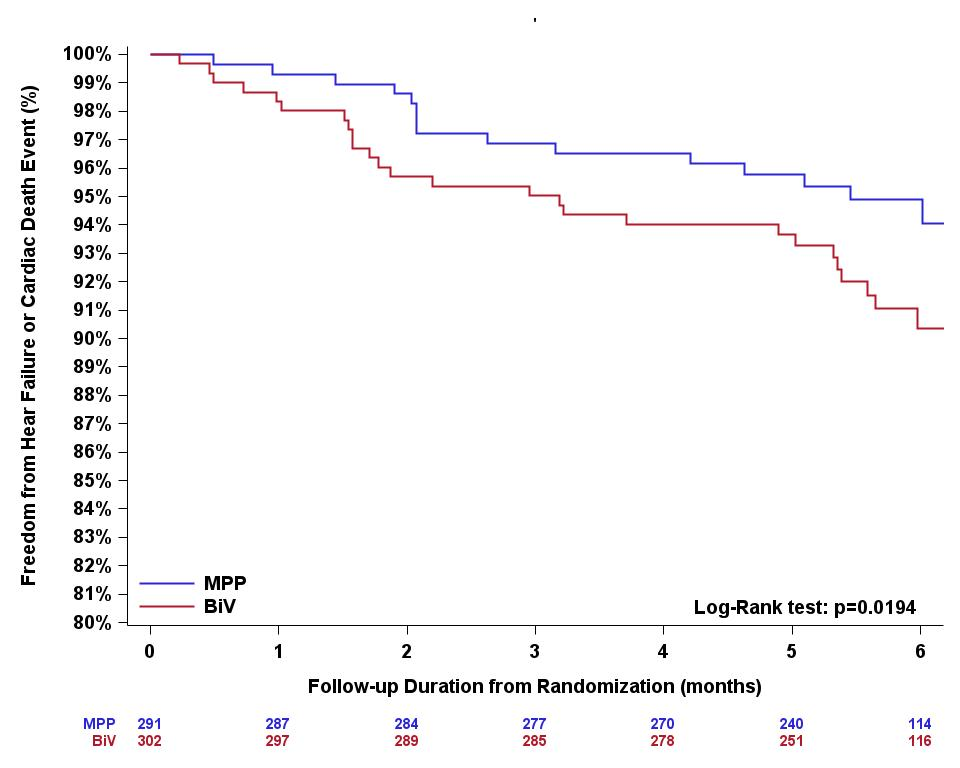


**Table A - CRT response rate (%) in MPP and BIV arms according to pre-specified patients characteristics (CRT response is defined as freedom from cardiac death or HF hospitalizations and as LV reverse remodeling)**

| **Variables** | **MPP**  **CRT Response (%)** | **BIV**  **CRT Response (%)** | **p value** |
| --- | --- | --- | --- |
| **All Patients (n=593)** | **33.0** | **23.5** | **0.010** |
| LBBB (n=292) | 33.8 | 27.9 | 0.275 |
| RBBB (n=49) | 28.6 | 9.5 | 0.102 |
| **IVCD (n=127)** | **31.0** | **14.5** | **0.025** |
| **Ischemic (n=320)** | **26.8** | **17.2** | **0.038** |
| Non-ischemic (n=273) | 38.8 | 30.9 | 0.172 |
| QRS < 150 ms (n=204) | 26.0 | 21.2 | 0.415 |
| **QRS ≥ 150 ms (n=340)** | **36.6** | **24.6** | **0.016** |
| NYHA II (n=301) | 29.7 | 30.1 | 0.949 |
| NYHA III/IV (n=291) | 35.0 | 16.9 | 0 |
| LVEF < 25% (n=250) | 32.0 | 24.2 | 0.173 |
| **LVEF ≥ 25% (n=343)** | **32.5** | **23.0** | **0.048** |
| **12M BIV % > 97% (n=409)** | **36.1** | **24.6** | **0.011** |
| LVESV < 150 ml (n=367) | 29.9 | 21.1 | 0.05 |
| LVESV ≥150.5 ml (n=226) | 36.0 | 27.7 | 0.181 |
| **Age < 69 years (n=308)** | **35.6** | **23.0** | **0.015** |
| Age ≥ 69 years (n=285) | 28.2 | 24.0 | 0.418 |
| Female (n=134) | 27.1 | 32.0 | 0.54 |
| **Male (n=459)** | **33.6** | **20.7** | **0.002** |
| QRS/LVEDV < 0.7811 (n=321) | 31.5 | 26.4 | 0.317 |
| **QRS/LVEDV ≥ 0.7811 (n=272)** | **33.3** | **20.3** | **0.015** |

MPP=MultiPoint Pacing; BIV=Biventricular Pacing; LBBB=Left Bundle Branch Block; RBBB=Right Bundle Branch Block; IVCD=Intraventricular conduction delay; NYHA=New York Heart Association; LVEF=Left ventricle ejection fraction; LVESV=Left ventricle end systolic volume; LVESV=Left ventricle end dyastolic volume

**Figure B - Effect of MPP programming on being a CRT responder in pre-defined patients subgroups (CRT response is defined as freedom from cardiac death or HF hospitalizations and as LV reverse remodeling)**

***Figure C – CRT response (proportion of patients with LVESV relative reduction >15%, between baseline and 6 months visit) as a function of interventricular electrical delay dispersion among the 4 LV dipoles in the whole study population***

*
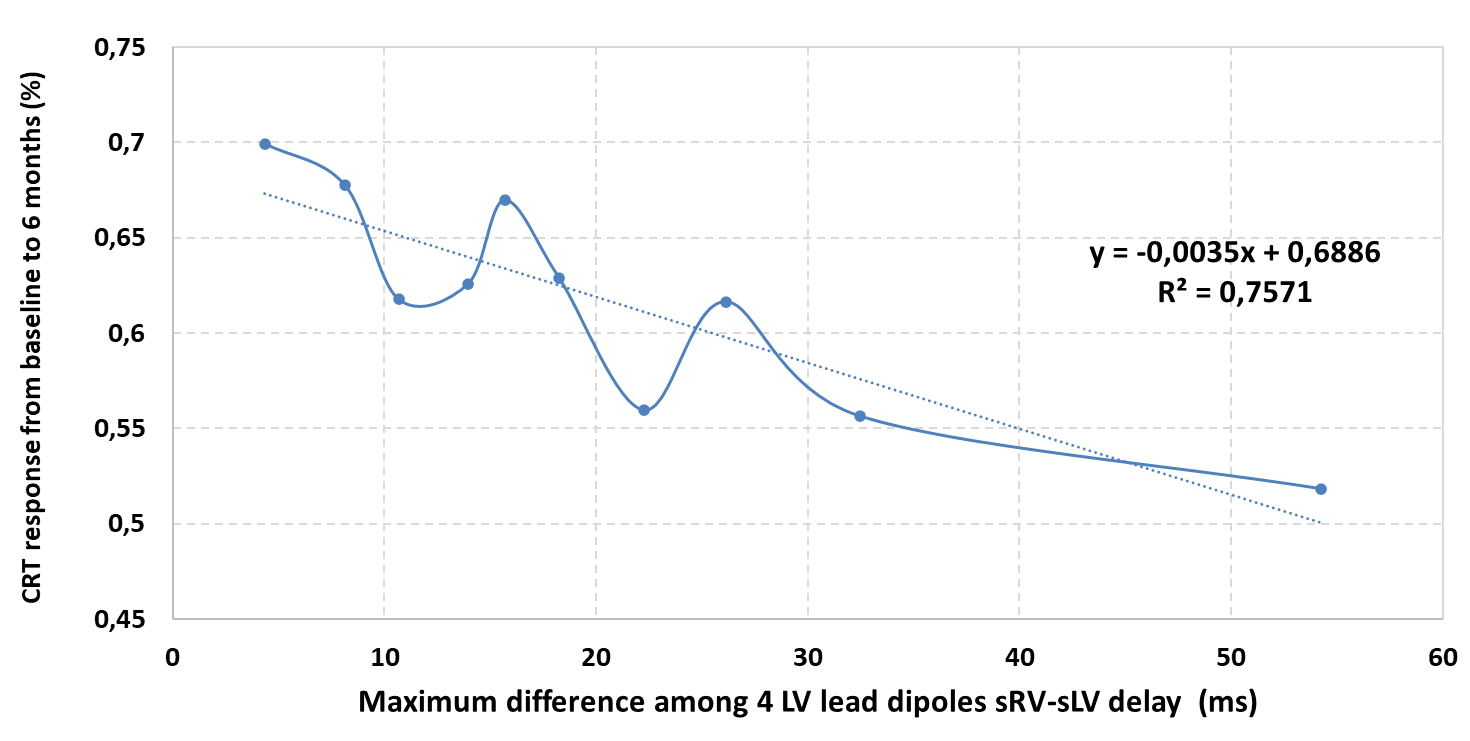
*

**Table B**

List of participating institutions and principal investigators in the MORE-CRT MPP trial.

| **Investigational Site** | **Principal Investigator** |
| --- | --- |
| Royal Adelaide Hospital | Sanders, Prash |
| The Alfred Hospital | Mariani, Justin |
| St. Andrews War Memorial Hospital | Hayes, John |
| Westmead Hospital | Sivagangabalan, Gopal |
| Klinik Floridsdorf | Achleitner, Reinhard |
| Wilhelminenspital Wien | Koch, Johannes |
| Krankenhaus der Stadt St. Polten | Thudt, Karin |
| Hopital Erasme | Casado, Ruben |
| St. Joseph Gilly | Leroy, Jean |
| Institut de Cardiologie de Montreal (Montreal Heart Inst.) | Thibault, Bernard |
| Rouge Valley Centenary | Janmohamed, Amir |
| St. Paul's Hospital | Chakrabarti, Shanta |
| Institut de Cardiologie de Quebec (Hopital Laval) | Molin, Franck |
| HSC, Eastern Health | Connors, Sean |
| QE II Health Sciences | Sapp, John |
| Kingston General Hospital | Simpson, Christopher |
| CHUM | Coutu, Benoit |
| McGill University Health Centre General Hospital | Essebag, Vidal |
| Royal Alexandra Hospital | Williams, Randall |
| St. Michael's Hospital | Mangat, Iqwal |
| Foothills Medical Centre | Sumner, Glen |
| CHUS Fleurimont | Ayala-Paredes, Felix |
| Prince of Wales Hospital | Chan, Joseph Yat Sun |
| Angiografia Clinica de Occidente | Dager Gomez, Antonio |
| Aalborg Sygehus Syd | Sogaard, Peter |
| Odense University Hospital | Johansen, Jens Brock |
| Skejby University Hospital | Nielsen, Jens |
| Turku University Hospital | Lund, Juha |
| Keski-Suomi Central Hospital | Nyman, Kai |
| CHRU Hopital de Pontchaillou | Leclercq, Christophe |
| CHRU Albert Michallon | Defaye, Pascal |
| CHRU Lille | Marquie, Christelle |
| Centre Cardiologique Du Nord | Piot, Olivier |
| Medipole Lyon-Villeurbanne | Poty, Herve |
| CHRU de Brest | Mansourati, Jacques |
| Hopital Clairval | Mechulan, Alexis |
| Hopital Pitie Salpetriere | Hidden-Lucet, Franciose |
| Hopital Prive du Confluent | Gras, Daniel |
| Hopital Saint Philibert | Guyomar, Yves |
| CHU - Montpellier | Pasquie, Jean-Luc |
| CHRU Rouen Hospital Charles Nicolle | Anselme, Frederic |
| CHU de Nancy - Hopital de Brabois | Blangy, Hugues |
| CHU Hopital G. & R. Laennec | Lande, Gilles |
| CHR de La Reunion - Site du CHFG | Clerici, Gael |
| Centre Hospitalier de Belfort-Montbeliard | Fouche, Renaud |
| Institute Cardio. Paris-Sud - Institut Jacques Cartier | Horvilleur, Jerome |
| CHRU Hopital de la Cote de Nacre | Pellissier, Arnaud |
| Hopital Saint Joseph | Gitenay, Edouard |
| CHU Rangueil Toulouse | Mondoly, Pierre |
| Centre Hospitalier de Valence | Miralles, Aurelien |
| CHU du Bocage | Laurent, Gabriel |
| CHU Gabriel Montpied | Eschalier, Romain |
| CHU Trousseau | Babuty, Dominique |
| Deutsches Herzzentrum Munchen des Freistaates Bayern | Kolb, Christof |
| Medizinische Hochschule Hannover | Veltmann, Christian |
| Medizinische Einrichtungen der Universitat zu Koln | Steven, Daniel |
| Berufsgenossenschaftliche Kliniken Bergmannsheil | Boesche, Leif |
| Herz-und Diabetes Zentrum NRW | Sommer, Philipp |
| Universitatsklinikum Leipzig AOR | Neef, Martin |
| Universitatsmedizin Berlin - Campus Benjamin Franklin (CBF) | Huemer, Martin |
| Evangelisches Krankenhaus Kalk gGmbH | Eberhardt, Frank |
| Schuchtermann-Schiller'sche Kliniken GmbH & Co. KG | Moennig, Gerold |
| Universitatsklinikum Munster | Eckardt, Lars |
| Klinikum Bielefeld gGmbH Klinikum-Mitte | Stellbrink, Christoph |
| Markische Kliniken GmbH Klinikum Ludenscheid | Lemke, Bernd |
| Klinikum Ingolstadt GmbH | Seidl, Karlheinz |
| Helios-Klinikum Erfurt GmbH | Schade, Anja |
| Klinikum Coburg GmbH | Brachmann, Johannes |
| Krankenhaus der Barmherzigen Bruder | Voss, Frederik |
| Universitatsklinikum Greifswald | Busch, Mathias |
| Herz- und Gefäßzentrum am Krankenhaus Neu-Bethlehem | Hansen, Claudius |
| Kerckhoff-Klinik gGmbH | Sperzel, Johannes |
| St.-Marien-Hospital GmbH | Perings, Christian |
| Kliniken der Friedrich-Alexander-Universitat | Arnold, Martin |
| Charite Campus Virchow Klinikum | Blaschke, Florian |
| Hegau-Bodensee-Hochrhein-Kliniken GmbH | Kollum, Marc |
| Albertinen-Krankenhaus Hamburg | Naegele, Herbert |
| Stadtisches Klinikum Gutersloh gGmbH | Er, Fikret |
| St. Vinzenz-Hospital | Winter, Stefan |
| Universitatsklinikum Wurzburg | Nordbeck, Peter |
| Universitatsmedizin Gottingen Georg-August-Universitat | Herting, Jonas |
| Kliniken Villingen-Schwenningen | Jung, Werner |
| Klinikum St. Georg gGMbH | Klein, Norbert |
| Elisabeth-Krankenhaus Essen GmbH | Schmitz, Dietmar |
| Klinikum Oldenburg gGmbH | Oswald, Hanno |
| Universitats-Herzzentrum Freiburg - Bad Krozingen | Restle, Christian |
| The Onassis Cardiac Center | Theodorakis, George |
| Escorts Heart Institute & Research Centre | Saxena, Anil |
| CARE Banjara | Narasimhan, Calambur |
| Medanta - The Medicity Hospital | Bhargava, Kartikeya |
| Care Institute of Medical Sciences | Naik, Ajay |
| Apollo Hospital | Karthigesan, Arumugam Murugesan |
| Pushpawati Singhania Hospital & Research Institute | Kler, Tarlochan |
| Medanta Medicity Hospital | Singh, Balbir |
| Fortis Hospital | Pal, Shashidhar |
| Postgraduate Institute of Medical Education & Research | Vijayvergiya, Rajesh |
| Asian Institute of Gastroenterology (AIG) Hospital | Narasimhan, Calambur |
| Sheba Medical Center | Bar Lev, David |
| Rabin Medical Center | Golovchiner, Gregory |
| Tel Aviv Medical Center | Viskin, Samuel |
| Hadassah - Ein Kerem | Luria, David |
| Casa di Cura Dpott. Pederzoli | Vicentini, Alfredo |
| Policlinico Casilino | Calo, Leonardo |
| Azienda Ospedaliero Universitaria Pisana | Bongiorni, Maria Grazia |
| Ospedale Giovanni Paolo II - Cardiology | Nicosia, Antonino |
| Ospedale Civile Maggiore di Verona Borgo Trento | Morani, Giovanni |
| Ospedale dei Pellegrini | Ducceschi, Valentino |
| Azienda Ospedaliera Di Venere | Bonfantino, Vincenzo |
| Az Osp.Universitaria Maggiore della Carita | Dell'era, Gabriele |
| Ospedale S. Giovanni Bosco | Capogrosso, Paolo |
| Ospedale di Portogruaro | Nangah, Rene |
| Presidio Osp. Vito Fazzi | Pisano, Ennio |
| Universita degli Studi di Padova | Bertaglia, Emanuele |
| Casa Sollievo della Sofferenza | Potenza, Domenico Rosario |
| Az. Osp. Spedali Civili di Brescia | Curnis, Antonio |
| Az.Osp.Universitaria Consorziale Policlinico | Favale, Stefano |
| Clinica Mediterranea | Iuliano, Assunta |
| Azienda Ospedaliera S.Anna e S.Sebastiano | Viscusi, Miguel |
| Policlinico S.Orsola Malpighi | Ziacchi, Matteo |
| Az. Osp.Sant'Anna | Russo, Giovanni |
| Ospedale Madre G. Vannini | Ansalone, Gerardo |
| Ospedale S. Giovanni Calibita Fatebenefratelli di Roma | Bianchi, Stefano |
| St. Marianna University School of Medicine Hospital | Harada, Tomoo |
| American University of Beirut Medical Center | Khoury, Maurice |
| Institut Jantung Negara | Razali, Omar |
| Mater Dei Hospital | Aquilina, Oscar |
| Isala - ZWolle | Delnoy, Peter-Paul |
| Amsterdam Academic Medical Centre (AMC) | Knops, Reinoud |
| Catharina Ziekenhuis | Houthuizen, Patrick |
| TweeSteden Ziekenhuis - Locatie Tilburg | Widdershoven, J.W.M.G. |
| Medisch Spectrum Twente | Van Es, Jan |
| Gornoslaskie Centrum Medyczne im.prof. Leszka Gieca | Wilczek, Jacek |
| Wojewodzki Specjalistyczny Szpital im. Bieganskiego | Bednarkiewicz, Zbigniew |
| Slaskie Centrum Chorob Serca | Kalarus, Zbigniew |
| Szpital Kliniczny Przemienienia Panskiego UM w Poznaniu | Mitkowski, Przemyslaw |
| Santa Maria Hospital | Marques, Pedro |
| Centro Hospitalar Vila Nova Gaia | Primo, Joao |
| Hospital de Braga | Rocha, Sergia |
| Hospital Garcia de Orta, EPE | Brand?o, Luis |
| Hospital de Santa Cruz | Adragao, Pedro |
| Centro Hospitalar do Alto Ave, Unidade de Guimarães | Sanfins, Victor |
| Heart Rhythm Management | Sotomonte Ariza, Juan |
| Hospital San Lucas Ponce | Perez, Francisco |
| Meshalkin National Medical Research Center | Romanov, Alexander |
| King Fahad Medical City | Al Samadi, Faisal |
| King Fahad Armed Forces Hospital | Bokhari, Fayez |
| National University Hospital | Seow, Swee Chong |
| Yonsei University Health System | Joung, Bo Young |
| Samsung Medical Center | Park, Seung-Jung |
| Sejong Hospital | Park, Mi Young |
| Seoul National University Bundang Hospital | Oh, Il Young |
| Seoul National University Hospital | Oh, Seil |
| Seoul St. Mary's Hospital | Oh, Yong-Seog |
| Hospital Universitario Infanta Cristina | Fernandez Concha, Joaquin |
| Hospital Universitari i Politecnic La Fe | Osca Asensi, Joaquin |
| Fundacion Jimenez Diaz | Sanchez Borque, Pepa |
| HCU Virgen de la Victoria | Alzueta Rodriguez, Javier |
| Hospital Ramon y Cajal | Hernandez Madrid, Antonio |
| Hospital Alvaro Cunqueiro, Dept of EP & Arrhythmias | Garcia Campo, Enrique |
| Hospital General Universitario Gregorio Marañon | Arenal, Angel |
| Hospital Universitario Puerta de Hierro | Toquero-Ramos, Jorge |
| Hospital de la Santa Creu I Sant Pau | Vinolas, Xavier |
| Hospital Universitario Doce de Octubre | Lopez-Gil, Maria |
| Hospital Universitario Virgen de la Nieves | Macias, Maria Rosa |
| Hospital Universitario A Coruña | Mosquera, Ignacio |
| Complexo Hospitalario Universitario de Santiago | Martinez Sande, Jose |
| Hospital Universitario Miguel Servet | Oloriz, Teresa |
| Karolinska University Hospital, Solna | Gadler, F |
| Hopital Cantonal Universitaire de Geneva | Burri, Haran |
| National Taiwan University Hospital | Ho, Li Ting |
| Chang Gung Memorial Hospital | Wang, Chun-Chieh |
| John Radcliffe Hospital | Betts, Tim |
| Golden Jubilee National Hospital | Gardner, Roy |
| The Royal Sussex County Hospital | Ellery, Sue Mary |
| Southampton University Hospital | Flett, Andrew |
| Manchester Heart Center, Manchester Royal Infirmary | Muhyaldeen, Sahrkaw |
| The Great Western Hospital | Foley, Paul |
| Queen Elizabeth Hospital | Leyva-Leon, Francisco |
| St. Thomas Hospital | Rinaldi, Christopher |
| Kings College Hospital | Scott, Paul |
| Wansbeck General Hospital | Runnett, Craig |
| Cardiac Rhythm Specialists, Inc. | Polosajian, Leo |
| Heart Center Research, LLC. | Jennings, John |
| Lancaster General Hospital | Bansal, Sandeep |
| Baptist Health Lexington | Tomassoni, Gery |
| Redmond Regional Medical Center | Styperek, Robert |
| Cardiovascular Associates of Mesa | Kaplan, Andrew |
| Colorado Heart & Vascular, P.C. | Venkataraman, Ganesh |
| Samaritan Heart & Vascular Institute - Cardiology Dept. | Hsing, Jeff |
| Glendale Adventist Medical Center | Mckenzie, John |
| St. Francis Hospital | Sellers, Matthew |
| Comprehensive Cardiovascular | Habib, Moksedul |
| EP Heart | Hariharan, Ramesh |
| Erlanger Medical Center | Manyam, Harish |
| Phoenix Cardiovascular Research Group | Bahu, Marwan |
| Baylor All Saints Medical Center at Fort Worth | Shah, Syed |
| Central Cardiology | Salvo, Jared |
| McLaren Health Care Corporation | Buerkel, Daniel |
| Memorial Katy Cardiology Associates | Kashani, Amir |
| Deborah Heart & Lung Center | Corbisiero, Raffaele |
| Scripps Health | Rogers, John |
| Vivek Mangla, MD | Mangla, Vivek |
| Coliseum Medical Centers | Hoffman, Jonathan |
| MidMichigan Medical Center-Midland | Islam, Nilofar |
| San Diego Cardiac Center | Athill, Charles |
| Brigham & Women's Hospital | Koplan, Bruce |
| Cardiology Associates of Fairfield County, PC | Tiano, Joseph |
| Cardiovascular Associates of the Delaware Valley | Levi, Steven |
| Eisenhower Medical Center | Feldman, Leon |
| Methodist University Hospital | Jha, Sunil |
| Sansum Clinic - Santa Barbara Medical Foundation | Cogert, Gregory |
| St. Elizabeth Medical Center - South Unit | Sinno, Mohamad |
| Munson Medical Center | Jaffe, Brian |
| CHI Health Creighton University Medical Center-Bergan Mercy | Abuissa, Hussam |
| St. Vincent Heart Clinic Arkansas | Chakka, Mangaraju |
